# The Cortical and Striatal Gene Expression Profile of 100 Hz Electroacupuncture Treatment in 6-Hydroxydopamine-Induced Parkinson's Disease Model

**DOI:** 10.1155/2012/908439

**Published:** 2012-01-26

**Authors:** Li-Rong Huo, Xi-Bin Liang, Bo Li, Jian-Tao Liang, Yi He, Yan-Jun Jia, Jun Jia, Xiao-Li Gong, Fen Yu, Xiao-Min Wang

**Affiliations:** ^1^Department of Physiology, Key Laboratory for Neurodegenerative Disorders of the Ministry of Education, Capital Medical University, Beijing 100069, China; ^2^Department of Neurology, Stanford University, Stanford, CA 94305, USA; ^3^Department of Neurosurgery, Xuanwu Hospital, Capital Medical University, Beijing 100053, China

## Abstract

Electroacupuncture (EA), especially high-frequency EA, has frequently been used as an alternative therapy for Parkinson disease (PD) and is reportedly effective for alleviating motor symptoms in patients and PD models. However, the molecular mechanism underlying its effectiveness is not completely understood. To implement a full-scale search for the targets of 100 Hz EA, we selected rat models treated with 6-hydroxydopamine into the unilateral MFB, which mimic end-stage PD. High-throughput microarray analysis was then used to uncover the regulated targets in the cortex and striatum after 4-week EA treatment. In the differentially regulated transcripts, the proportion of recovered expression profiles in the genes, the functional categories of targets in different profiles, and the affected pathways were analyzed. Our results suggested that the recovery of homeostasis in the transcript network and many regulated functional clusters in the cortex and striatum after EA treatment may contribute to the behavioral improvement of PD rats.

## 1. Introduction

Parkinson's disease (PD) is an age-related neurodegenerative disease that affects about 1–3% of the population over 65 years of age and is characterized by the progressive loss of DAergic neurons in the substantia nigra pars compacta and an associated decline in striatal dopamine [1]. It is known that “idiopathic” PD (>85% of all cases) does not appear to exhibit heritability [2]. In addition, there is no cure for PD, and the underlying pathogenesis of the disease is still unknown [2]. In general, we understand that the pathology of PD is complex and is most likely a “consequence of an unspecified combination of genetic and environmental factors, which induce a common pathogenic cascade of molecular events” [3]. Pharmacological and surgical therapies are available that can alleviate some of the symptoms, but these interventions are associated with serious side effects and generally lose their efficacy over time [4]. Acupuncture, however, has long been known to have therapeutic effects on chronic and acute pain. Today, electro-acupuncture (EA) is frequently used as an alternative therapy for PD and reportedly leads to subjective improvements in PD patients [5, 6]. An increasing number of clinical studies and experimental data support EA, especially high-frequency EA, as an effective therapy for alleviating motor symptoms in patients and PD models [7–9]. However, the underlying neuroprotective mechanisms of acupuncture treatment in PD are not yet understood. Some experimental results reveal that EA could increase the number of tyrosine hydroxylase- (TH-) positive neurons in the substantia nigra and striatum (STR) of an MPTP- (1-methyl-4-phenyl-1,2,3,6-tetrahydropyridine-) treated mouse model [10, 11]. Using the hemiparkinsonian rat model induced by unilateral transection of the medial forebrain bundle (MFB), our previous studies showed that 100 Hz EA could significantly improve the apomorphine-induced motor disorder symptoms, but the dopamine (DA) content did not increase significantly [12, 13]. Moreover, the effects of high-frequency EA on the motor symptoms of Parkinsonian rats involved the upregulation of endogenous neurotrophins [8, 9] and BDNF and trkB receptors in a 6-hydroxydopamine- (6-OHDA-) induced rat PD model [14], as well as the restoration of the homeostasis of DAergic transmission in the basal ganglia circuit and the suppression of inflammatory responses in the ventral midbrain [9, 12, 15]. These results suggest that the mechanisms underlying the benefits of acupuncture are systemic and unknown. Thus, it is especially important to find EA-targeted transcripts. Gene expression profiling using microarray technology could reveal the complex nature of disease genesis and progression [16] and the many targets of certain therapeutic measures at the genomic level [17]. To search for EA-affected transcripts after 6-OHDA treatment and obtain insight into the potential mechanism of EA's effect, we applied the microarray method to explore gene expression profiles in a control group, a 6-OHDA-unilateral lesioned rat model, and an EA-treated group for 4 weeks after 6-OHDA lesion. We observed the differential genes at the transcription level and the changed gene profiles in the cortex and STR.

## 2. Materials and Methods

### 2.1. Animal Care and Unilateral MFB Injection with 6-OHDA

Adult female Sprague Dawley rats weighing 200–220 g were obtained from the laboratory animal center, Capital University of Medical Sciences, and housed in a standard 12-h on/off light cycle with ad libitum access to food and water. The experimental procedures were approved by the Committee on Animal Care and Usage, Capital Medical University, and all efforts were made to minimize animal suffering. 

The artificial lesion with 6-OHDA into the MFB is considered an appropriate model to study late-stage PD [18]. After adapting to their surroundings for 3 days, SD rats were anesthetized with chloral hydrate (350 mg/kg, i.p.) and then fixed on a stereotaxic frame (David Kopf Instruments, Tujunga, CA, USA) with the mouth bar set at −3.3 mm. The skull was exposed, and a burr hole was drilled to introduce a syringe for a single injection of the 6-OHDA solution (containing 2 *μ*g 6-OHDA per *μ*L in 0.01% ascorbicacid, pH = 5). To minimize variability due to degradation of the toxin, the 6-OHDA solutions were freshly made, kept on ice, and protected from exposure to light. The solution was injected into the left MFB according to the atlas of König and Klippel (1963); the coordinates relative to bregma were ML: −2.5 mm, AP: −3.8 mm, and DV: −8.1 mm. A total volume of 4 *μ*L 6-OHDA was injected at a flow rate of 1 *μ*L/min, and the syringe was left in place for 2 min and then slowly removed over a 1-2 min time period. The skin was sutured, and then the animals received 4 mg/kg ketofen i.p., as an analgesic and were allowed to recover before returning to the animal housing facilities. The lesion was allowed to progress for 5 weeks, after which the animals were sacrificed for postmortem analyses.

### 2.2. Behavioral Testing

**Figure 1:**
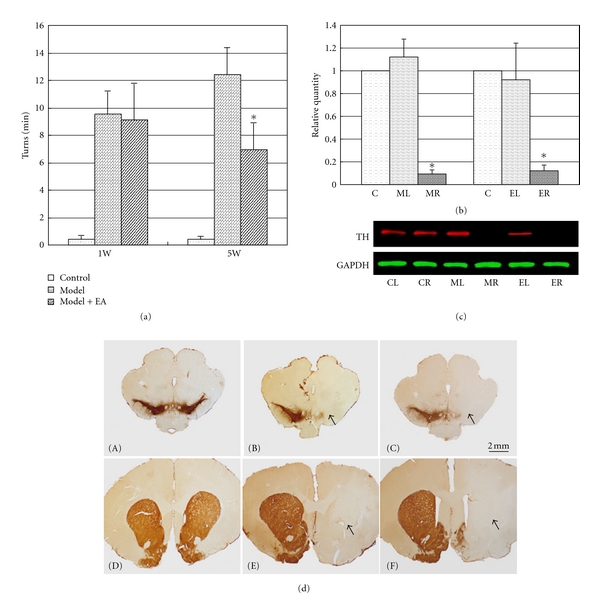
PD rat models were prepared using 6-OHDA and were treated with 100 Hz EA. EA at 100 Hz was applied at the acupuncture points DAZHUI (GV14) and BAIHUI (GV21) for 28 days. Rotational behavior induced by apomorphine was measured 1, 2, and 5 weeks after 6-OHDA treatment of the MFB. (a) The *Y*-axis represents the net number of turns of rats per minute. The *X*-axis represents the detection time. The net number of turns per minute of the 5-week EA-treated group decreased significantly compared to the corresponding 5-week model group (*P* < 0.001). ((b) and (c)) Western blotting results revealed that the expression of TH was significantly downregulated in the right STR of the 6-OHDA-treated rats (MR) and the EA-treated model rats (ER) compared to the nonlesioned side (c). CL and CR represent the left and right STR of the control rats, respectively. The relative grey level is presented in (b) (*P < 0.01). The *Y*-axis represents the relative grey value versus the control. The *X*-axis represents different groups. In addition, the extensive loss of DA neurons in the rat SN and STR was detected using Tyrosine hydroxylase- (TH-) specific immunostaining (scale bar 2 mm, (d)-(A)–(d)-(F)). As indicated by the arrows in (d), representative microphotographs of the right STR ((d)-(E) and (d)-(F)) and SN ((d)-(B) and (d)-(C)) after TH immunostaining showed an extensive reduction in the density of TH-immunoreactive axons in the STR and SN of 6-OHDA-treated rats, regardless of EA treatment (right STR of (d)-(E) and (d)-(F) and right SN of (d)-(B) and (d)-(C)), compared to the control ((d)-(A) and (d)-(D) or the left of model STR or SN).

Rotational testing was performed blindly and in automatic rotameter bowls (Columbus Instruments, USA) prior to MFB lesion and 7 (one week), 14 (two weeks), and 35 days (5 weeks) following the lesion (Figure 1(b)), a method previously reported by Liang et al. [9]. Changes in rotational behavior were assessed by monitoring body rotations induced by apomorphine (0.5 mg/kg, s.c.). The net number of rotations (contralateral-ipsilateral) was recorded over a time span of 45 min, and the number of turns per minute was calculated [13, 14, 19]. This behavioral test was performed blindly. Data were reported as means ± SEM. Statistical significance was assessed using one-way ANOVA followed by the Newman-Keuls post hoc test of differences between groups. A *P* < 0.01 was considered statistically significant in this portion of the experiment.

### 2.3. High-Frequency EA Stimulation

EA stimulation at 100 Hz was performed following our previously described method [8, 9, 12]. Rats were randomly divided into three groups: the sham group, the 6-OHDA-injected group, and the 6-OHDA-injected group with EA stimulation at 100 Hz. Animals in the sham group underwent the same operation without injection of 6-OHDA. EA stimulation was administered from day 8 following the 6-OHDA injection after behavioral testing at one week. Two stainless steel needles 0.25 mm in diameter and 5 mm in length were inserted obliquely at the acupuncture point DAZHUI (GV 14, directly below the spinous process of the vertebra prominens) and horizontally at BAIHUI (GV 21, at the midpoint of the line connecting the two ears). Bidirectional square-wave electrical pulses (0.2 ms duration), designated EA, were administered for a total of 30 min each day, 6 days per week. The duration of EA treatment was limited to 4 weeks. The intensity of the stimulation was increased stepwise from 1 to 2 mA and then to 3 mA, with each step lasting for 10 min. The animals remained in the cage during EA administration in an awake, unrestrained condition.

### 2.4. Immunohistochemical Analysis

Rats were deeply anesthetized with 450 mg/kg chloral hydrate and then transcardially perfused with 100 mL saline followed by 200 mL 4% paraformaldehyde in phosphate buffer. Brains were dissected and postfixed in the same fixative and cryoprotected in 30% sucrose solution for 3–5 days. Frozen sections were cut into 30 *μ*m thick sections on a cryostat and processed for TH-immunohistochemistry. Every sixth section of the SN or STR was selected from each brain. After several washes, brain slices were incubated with TH antibody (diluted 1 : 2000; Chemicon, USA) for 24 h at 4°C. The antibody was detected using an ABC Elite kit (Vector laboratories, USA) with 3, 3′-diaminobenzidine (DAB) and nickel enhancement [13].

### 2.5. Protein Extraction and Western Blot

To obtain protein extracts, rats were sacrificed by cervical dislocation 2 days after the last electroacupuncture treatment, and each encephalic region was dissected rapidly and frozen using liquid nitrogen and kept at −80°C until use. The frozen substantia nigras were homogenized 4 times with sonication at 70% intensity in 10 s bursts with 500 *μ*L of routine SDS-protein lysing buffer (including protease inhibitor cocktail, P2714, Sigma). The protein concentration of the final extract solution was determined using the BCA method. The western blotting procedures followed the standard protocol. Protein lysates were separated by electrophoresis and transferred to nitrocellulose membranes. The membranes were blocked with 5% nonfat dry milk in PBS containing 0.2% Tween 20 for 1 h and then incubated overnight at 4°C with the indicated primary antibody. Membranes were then treated with IRDye  800 (green) or IRDye  700 (red) conjugated affinity purified anti-mouse, anti-rabbit secondary IgG for 1 h, followed by three washes with PBS containing 0.1% Tween 20 and two washes with PBS alone. The fluorescent bands were visualized using an LI-COR Odyssey infrared double-fluorescence imaging system (American Company LI-COR). 

### 2.6. Sample Preparation and Microarray Image Analysis

Total RNA was separately extracted from the nine individual samples using the RNeasy Mini Kit (QIAGEN, 74106). Microarray analysis was performed and repeated 3 times using a biological sample in each group with the Whole Rat Genome 4 × 44 K microarrays (Gene expression hybridization kit, 5188-5242, Agilent). Following the protocols of the Low RNA Input Linear Amplification and Labeling Kit Plus (Agilent, #5184-3523), we synthesized double-stranded cDNA and applied it as a template to label cRNA. For each hybridization, 2 *μ*g of total RNA from each sample (control or model) was used. Each sample cRNA (labeled with Cy3 (Cy3 NHS ester, PA13105 GE healthcare)) was individually hybridized on microarrays. The labeling and hybridization steps were carried out according to the Agilent protocol, and the images were scanned using a microarray scanner (Agilent, G2565BA). 

### 2.7. Mul-Class Dif Analysis

We applied the RVM *F*-test to filter the differentially expressed genes in the control and experiment group because the RVM *F*-test can effectively increase the degrees of freedom in cases of small samples. After the significance analysis and FDR analysis, we selected the differentially expressed genes according to the *P* value threshold [20, 21]. 

### 2.8. STC Analysis

The series test of cluster (STC) algorithm of gene expression dynamics was used to profile the gene expression time series and to identify the most probable set of clusters generating the observed time series. This method explicitly took into account the dynamic nature of the temporal gene expression profiles during clustering and identified the number of distinct clusters [22, 23]. 

We selected differential expression genes in a logical sequence according to RVM (Random variance model) corrective ANOVA. In accordance with the different signal density change profiles of genes under different conditions, we identify a set of unique model expression profiles. The raw expression values were converted into log_2   _ratios. Using a strategy for clustering short time-series gene expression data, we defined a set of unique profiles. The expression model profiles are related to the actual or expected number of genes assigned to each model profile. Significant profiles have higher probability than expected by Fisher's exact test and multiple comparison test [24].

### 2.9. STC-Gene Functional Annotation

For transcripts in each profile of STC analysis, functional annotation and classification were completed using DAVID 6.7 b (current release, Jan. 2010) of DAVID Bioinformatics Resources (http://david.abcc.ncifcrf.gov/) [25, 26]. Functional annotation is applied to the genes belonging to certain specific profiles. It is used to find the main function of the genes with the same expression trend. Gene ontology (GO) is a key functional classification of DAVID.

### 2.10. Dynamic-GeneNet (Coexpression Network)

We also construct gene coexpression networks to find the interactions among genes in selected STC profiles (Figure 3(a), (A)–(F)) [27]. Gene coexpression networks were built according to the normalized signal intensity of specific expression genes. For each pair of genes, we calculated the Pearson correlation and chose the significant correlation pairs to construct the network (Figure 1S) [28]. 

 In the network analysis, the degree centrality is one of the simplest and most important measures of the centrality of a gene and its relative importance within a network. Degree centrality is defined as the number of links that one node has to all of the other nodes [29]. Moreover, in studying some properties of the networks, k-cores in graph theory were introduced as a method of simplifying the analysis of graph topologies. A k-core of a network is a subnetwork in which all nodes are connected to at least k other genes in the subnetwork. A k-core of a protein-protein interaction network usually contains cohesive groups of proteins [29].

The purpose of Network Structure Analysis is to locate core regulatory factors (genes), in one network. Core regulatory factors connect the most adjacent genes and have the largest degrees. When considering different networks, the core regulatory factors were determined by the degree of difference between two class samples [30]. The core regulatory factors always have the largest degree differences. The distinct figures of our coexpression network comprising the selected genes are shown in Figures S1A and B.

### 2.11. Pathway Analysis

Similarly, pathway analysis was used to determine the significant pathway of the differential genes according to KEGG, Biocarta, and Reatome. We used Fisher's exact test and the chi-square test to select the significant pathway, and the threshold of significance was defined by the *P* value and FDR. The enrichment was calculated as previously described [31–33].

### 2.12. Clustering and TreeView for Identified Transcripts

Hierarchical clustering was performed in Cluster 3.0 soft. For nine samples (Cortex or STR), a list was prepared of the selected genes to be clustered. The normal signal data for selected genes were adjusted to become log transform data. Then the data were arranged according the requirements of Cluster 3.0 and “median” was selected for center genes and arrays. After that, the results of clustering of selected genes were presented by Java TreeView and exported to the images.

### 2.13. Selected Gene Expression Changes Were Validated Using qRT-PCR

Total RNA was extracted from the cortex or STR using a standard trizol (Invitrogen, 15596-018) procedure from each tissue sample. RNA was treated with RNase-free DNase I, and 3 *μ*g of total RNA was subjected to cDNA synthesis with M-MLV reverse transcriptase (Promega, M170) in a 30 *μ*L liquid phase reaction for RT-PCR, from which 1 *μ*L of cDNA was used for PCR amplification. The primers used as an internal control for RT-PCR to amplify human 18 S were 5′-GGAAGGGCACCACCAGGAGT and 5′-TGC AGCCCCGGACATCTAAG. The primers used for amplifying 6-OHDA-targeted RNAs in real-time PCR (Stratagene MX3000P Sequence Detection system, USA) are listed in Table S1 (Supplementary Materials). The trials were completed by SYBR  Premix Ex Taq  kit (Takara, DRR041B). The PCR protocol consisted of 1 min at 95°C followed by 40 cycles of 30 s at 95°C, 30 s at 55°C, and 1 min at 72°C (Figure 4(b)). The levels of target gene expression were quantified relative to the level of 18 S using the standard curve method. The specificities of RT-PCR products were confirmed by the presence of both a single dissociation curve for the product and a single band, with corresponding molecular weight revealed by agarose gel electrophoresis. The statistical significance of the cluster gene rate and the discrete rate was assessed using *t*-test. A *P* value < 0.05 was considered statistically significant.

## 3. Results

### 3.1. EA at 100 Hz Attenuated the Rotational Behavior in a 6-OHDA PD Model

As seen in Figure 1, a nearly complete denervation of the SN in a rat model was observed in our experiments (Figures 1(d)-(B) and 1(d)-(E)). As hypothesized, the high-frequency EA stimulation did not to prevent the reduction in TH-positive dendritic fibers in the lesion-lateral SN and STR (Figures 1(d)-(C) and 1(d)-(F)). However, EA stimulation at 100 Hz reduced the number of rotations exhibited by the unilaterally 6-OHDA lesioned PD models (Figure 1(a)). The rotational behavior induced by apomorphine (0.5 mg/kg) was measured 1 (7 days), 2 (14 days), and 5 weeks (35 days) after the 6-OHDA-treatment of the MFB. The net number of turns was calculated as the number of contralateral turns minus the number of ipsilateral turns. The values are expressed as means ± SEM (*n*  = 10–14 per group, *P* < 0.01 between model/EA group and control). The turns/min of the control group was 0.42 ± 0.28 turns/min for 1 week, 0.33 ± 0.12 turns/min for 2 weeks and 0.43 ± 0.19 turns/min for 5 weeks. The rats in the 6-OHDA-treated group exhibited greater rotational asymmetry in the direction contralateral to the lesion (9.52 ± 1.69 turns/min at 1 weeks; 8.24 ± 2.10 turns/min at 2 weeks; and 12.44 ± 1.94 turns/min at 5 weeks) compared to the rats in the EA-treated group (9.13 ± 2.65 turns/min for 1 week; 7.88 ± 2.30 turns/min for 2 weeks; 6.92 ± 1.98 turns/min for 5 weeks). The number of turns of the 5-week EA-treated group decreased significantly compared to the corresponding 5-week model group (*P* < 0.001, the results from week 1 and week 5 are presented in Figure 1(a)). These results revealed that the number of apomorphine-induced rotations exhibited by 6-OHDA PD rats is reduced after EA treatment, although a histological recovery to normal levels could not be identified. 

### 3.2. EA at 100 Hz Caused a Multimolecular Change In Vivo

**Figure 2:**
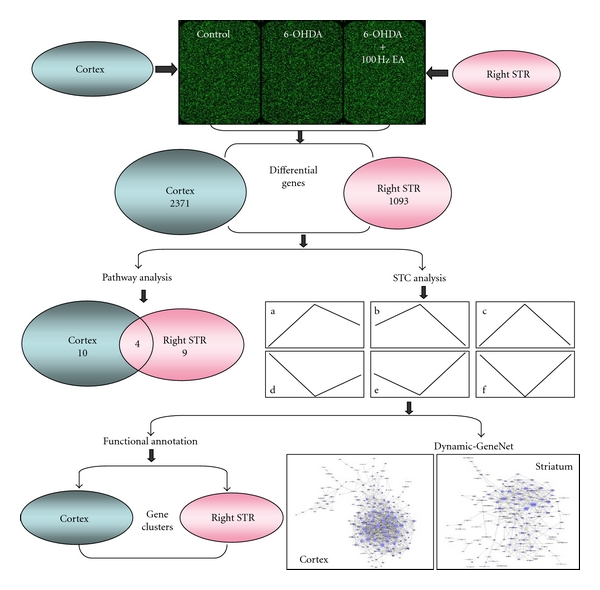
The flow-sheet was used for the data analysis of genomic profile. Three biology duplicate samples in each group were used for microarray analyzing. The cortex and right STR were examined, and the differential genes in this figure came from comparison of three groups (*P* < 0.05). To observe these genes in depth, STC (Figure 3) and pathway analysis were performed. Dividing differential genes into groups according to 16 profiles by STC, we then completed a functional annotation (Tables 1–3, S1–4). Then, genes from the 6 types of recovery expression profiles (A)–(F) after EA-treatment were subjected to Dynamic-GeneNet analysis. The genes of three profiles (STC (a), (b), and (c)) were the principal part in the spatial network.

**Figure 3:**
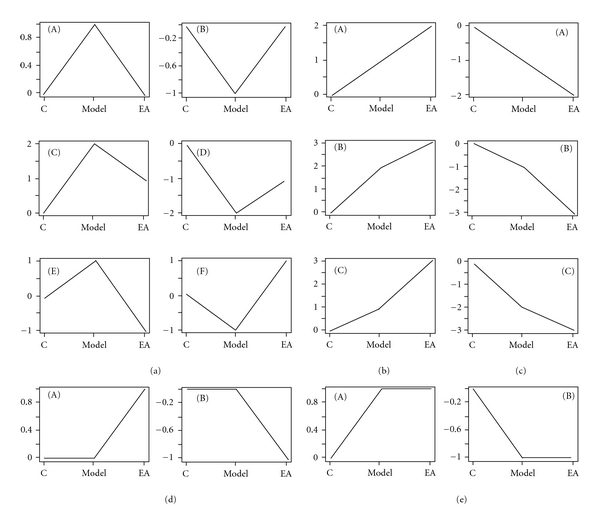
STC analyzed for differential genes in the cortex and STR after 6-OHDA or 6-OHDA/EA treatment. There were 16 types of profiles for differential genes in three experimental groups of the cortex or STR (control, model, and EA group). The number on the *Y*-axis is the degree of change and not a concrete value. These profiles can be divided into 5 subgroups. (a) Recovery expression profiles group ((a), (A)–(F)). After EA treatment, these genes tended to recover the normal level. Genes of (a)-(A) and (B) were completely recovered. Genes of (a)-(C) and (D), however, were only partially recovered. (a)-(E) and (F) represent the overcorrect genes. (b) Upregulated group ((b), (A)–(C)). These genes continued to be upregulated after EA treatment. (c) Downregulated group ((c), (A)–(C)). These genes continued to be downregulated after EA treatment. These two part of genes ((b) and (c)) need to be considered individually and they belong to the portion of genes regulated by EA. (d) This group was only regulated by EA ((d)-(A) and (d)-(B)). These genes were up- or downregulated only after 100 Hz EA treatment. (e) The group continued to maintain the model group level. These genes displayed no change after EA treatment but were upregulated or downregulated compared to control. A detailed functional annotation is displayed (see Tables S2–8 in supplementary material available on line at doi:10.1155/2012/908439) and it shows that the target genes of 100 Hz EA are extensive and involved in multifunctional regulation.

An ANOVA for the three groups (Control, Model and EA group) in our studies revealed that there were 2371 differential genes in the cortex and 1093 differential genes in the STR (*P* < 0.05, Figure 2). After the STC analysis, these differential genes were divided into 16 types of expression profiles according to the alignment of the control, model, and EA-treated groups (Figure 3). The transcripts in profile (a) showed that the effect of EA in 6-OHDA lesioned animals return to the proximalis levels of gene expression that were observed in the control group. The functional annotations for these transcripts in the 16 profiles refer to Tables S2–8 (Supplementary Material). Among the results of the functional annotations, the majority of the regulated genes belonged to profile (a) (recovered), (b) (upregulated), (c) (downregulated), or (e) (sustained) after EA treatment. The functions of these transcripts were found to be involved in many aspects, which were presented in supplementary Tables S2–5 and Tables S7–8, after DAVID 6.7 analysis. Therefore, 100 Hz EA may cause a multimolecular change in vivo in a PD model.

In addition, our results reveal that there were 255 mutual genes, with 16 types of profiles between the cortex and STR, and that 87.06 percent of these genes (222/255) were in the noncontradicted profiles (profiles in addition to the opposite regulated genes by EA). After the DAVID 6.7 analysis, 74.36 percent of the genes (29/39) listed in Table S9, which had names and functional annotations, belonged to the genes in profile (a) of Figure 3. After the functional annotation for the genes in Table S9, the categories of “cognition and sensory perception,” “olfactory transduction and receptor activity,” “G-protein coupled receptor protein signaling pathway,” “integral to membrane,” and “neurological system process” were found to be regulated by high-frequency EA (*P* ≤ 0.01, Table S10). Multiple transcripts of these functional categories trended toward recovery to normal levels (Table S9).

The identification of the significantly regulated genes (the log_2_ difference absolute ≥ 1 compared to model, *P* < 0.05) after EA treatment was important to explain the internal changes to disorder and turnover of the genes. According to the standard in the previous bracket, 71 genes in the cortex were selected, and 50 genes, or 70.42% (50/71), were in profile (a) of Figure 3 (Table S11). In addition, 38 genes in the STR were selected, of which 78.95%, or 30 genes, were in profile (a) of Figure 3. The notations of these genes are listed in Table S11. This table shows which transcripts belong to each profile.

### 3.3. Multipathways Targeted by 100 Hz EA

The following ten pathways in the cortex and nine pathways in the STR were predominantly affected by 100 Hz EA (*P* < 0.05, Table S12): the olfactory transduction pathway (*P* < 0.001), Alzheimer's disease pathway (*P* < 0.001), Huntington's disease pathway (*P* < 0.001), neuroactive ligand-receptor interaction pathway (*P* < 0.001), inositol phosphate metabolism pathway (*P* < 0.01), calcium signaling pathway (*P* < 0.05), systemic lupus erythematosus pathway (*P* < 0.05), amyotrophic lateral sclerosis (ALS) pathway (*P* < 0.05), p53 signaling pathway (*P* < 0.05), and long-term depression pathway (*P* < 0.05) in the cortex; the neuroactive ligand-receptor interaction pathway (*P* < 0.01), olfactory transduction pathway (*P* < 0.01), arachidonic acid metabolism pathway (*P* < 0.01), amyotrophic lateral sclerosis (ALS) pathway (*P* < 0.05), long-term depression pathway (*P* < 0.05), ECM-receptor interaction pathway (*P* < 0.05), taurine and hypotaurine metabolism pathway (*P* < 0.05), cytokine-cytokine receptor interaction pathway (*P* < 0.05), and MAPK signaling pathway (*P* < 0.05) in the lesion-lateral STR (Table S12).

**Figure 4:**
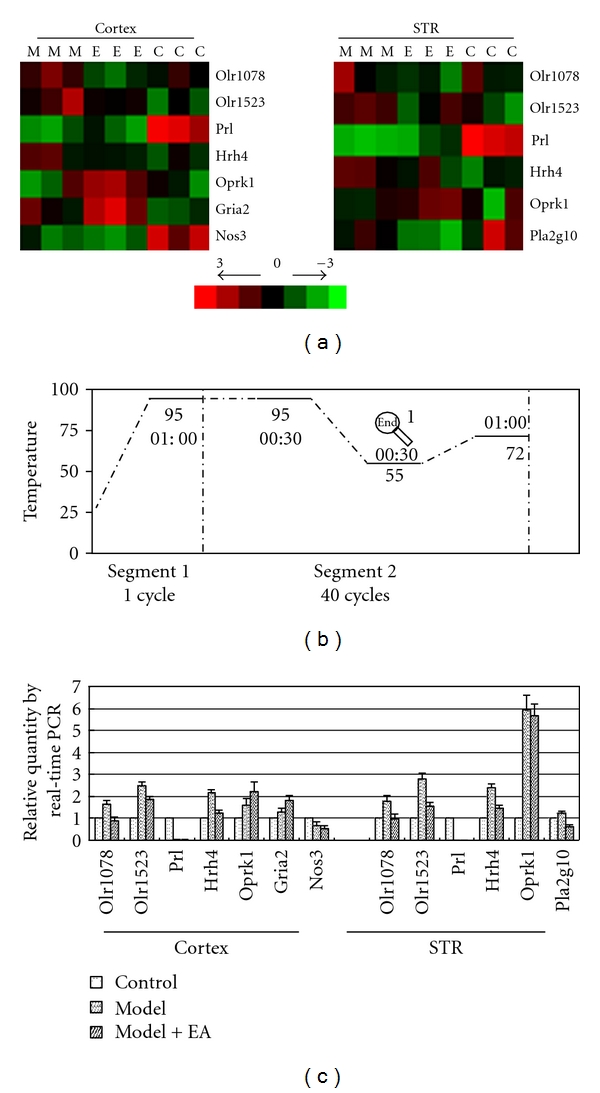
Selected transcripts clustered and validated with real-time PCR. After pathway analysis, we selected genes involved in major pathways (the common pathways in the cortex and STR, Table  7). First, selected genes were clustered using Cluster 3.0 according to the LOG value in the STR groups (a) Red is relatively upregulated and green is relatively downregulated in different samples. The three “Cs” are the three control samples. The three “Ms” are the model samples. The three “Es” are the EA-treated samples. Then, to verify the reliability of the microarray analysis, we verified these selected genes from the clustering diagram using real-time RT-PCR. The real-time PCR primers are listed on Table S1 (devised and synthesized by Takara). The reaction procedure was present on (b). If the level of control was regarded as 1, the corresponding gene expression in model and EA-treated groups were present on (c). These transcripts analyzed here showed coherent profiles with cluster (a).

To validate the microarray results, we selected several transcripts to cluster (Figure 4(a)) that were involved in major pathways (Table S12) and validated the clustering result using real-time PCR (Figure 4(c)). The functions of the protein products for these transcripts are listed in Table S13. To some extent, the identified results confirmed the reliability of the array analysis.

### 3.4. Internal Molecule Network Targeted by 100 Hz EA

**Figure 5:**
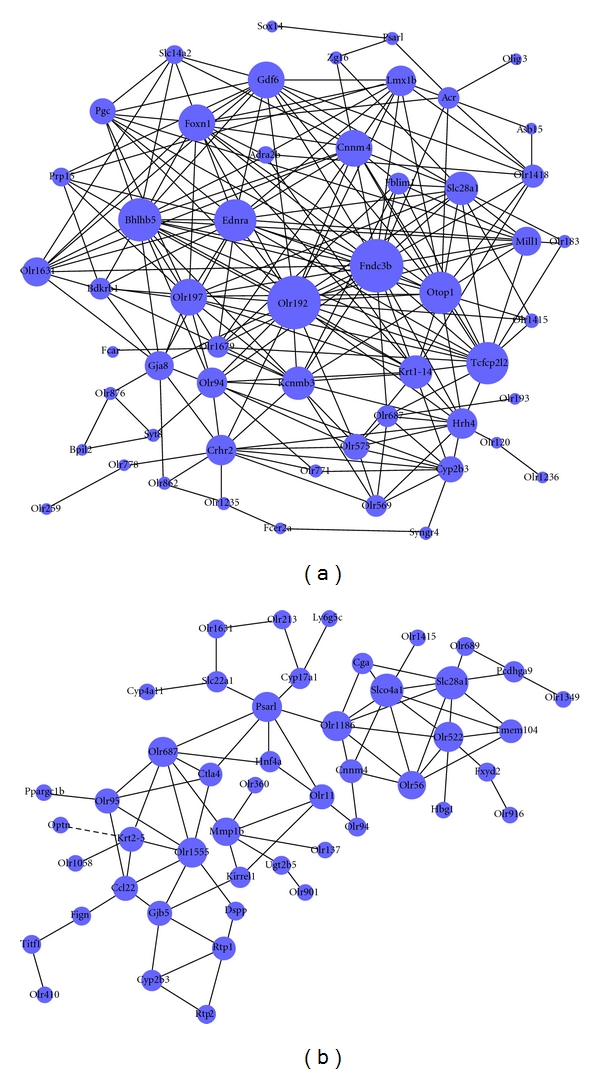
Simplified dynamic gene network for genes in profile of Figure 3(a). The global dynamic gene networks of the cortex and STR are shown in Figures S1A and S1B. A simplified network is presented here with the genes without names or some minor genes in the network deleted. The blue dots indicate the differentially regulated genes. The lines show the relationship between genes. The solid line denotes positive regulation, and the dashed line denotes negative regulation. The size of the dots indicates the capability of the gene to interact with others, quantified by “degree” (refer to Section 2). The larger the degree, the more the genes interacted with the corresponding genes and the more important this gene was in the network.

The integrity coexpression network for the six recovered profiles of the cortex and STR is presented in Figure S1. The simplified network is shown in Figure 5 (genes without names or with poor interactions were deleted). Combining these figures, the network density and nodes' degrees suggested that the cortex assumes a more important role in the regulation of internal balance after EA treatment. As seen in Figure S1 and Figure 5, FAD104 (FNDC3B, XM___226988, degree = 42), olfactory receptor 192 (OLR192, NM___001000549, degree = 40), basic helix-loop-helix domain containing class B5 (BHLHB5, XM___345190, degree = 32), otopetrin 1 (OTOP1, NM___181433, degree = 32), and endothelin receptor type A (ENDRA, NM___012550, degree = 31) are the core nodes in the cortex network. Mitochondrial protease presenilins-associated rhomboid-like protein (PARL/PSARL, ENSRNOT00000030837, degree = 20), olfactory receptor 522 (OLR522, NM___001000562, degree = 18), and member 4a1 of the solute carrier organic anion transporter family (SLCO4A1, NM___133608, degree = 15) are the most important nodes in the impaired STR network.

## 4. Discussion

Acupuncture is a popular alternative therapy in patients with Parkinson's disease (PD) and may provide benefit in the clinical setting [34]. Although acupuncture has not been used to cure PD, a survey of PD patients demonstrated that patients who received acupuncture reported an improvement in their symptoms [7]. In addition, acupuncture is very safe and well tolerated in clinical practice. However, the mechanisms underlying the neuroprotective effects of this technique are not yet clear.

The best course of PD therapy may be to increase the DA content of the striatum. However, increasing evidence suggests that a poor relationship exists between the dopamine content of the striatum and the improvement of motor symptoms during PD treatment [35, 36]. Recently, Park et al. reported that the mechanism of acupuncture involves multiple factors [10, 11, 17]. In addition, it has been argued that DA neurons and other cells within the SN and adjacent brain regions are involved in PD pathology [37, 38]. Our results and those of work aiming to quantify the effects of EA demonstrate that the effects may be produced by the coordination of several internal brain regions including cortex and STR.

To treat the neurochemical phenomenon of dopamine depletion, we aimed to identify the molecular effects of EA. A rodent model with 6-OHDA injected into the MFB mimics end-stage PD [18]. The number of rotations exhibited by an animal after amphetamine administration can be used to distinguish between partial and near complete (>90%) denervation in the SN [18]. Thus, a nearly complete denervation in the SN of a rat model was observed in our experiments (Figure 1(d)). Previously, we demonstrated that long-term, high-frequency EA stimulation on the points of DAZHUI (GV 14) and BAIHUI (GV 21) attenuated the rotational behavior of PD rats with partial lesions and improved motor activity [8, 9, 12]. In addition, previous results suggest that the effects of EA may be multitargeted and may be effective at PD without increasing dopamine (DA) [12, 13]. Thus, the 6-OHDA-injected rat model may provide an opportunity to test how the cortex and lesion-lateral STR develop a concerted reaction and change after EA treatment. High-frequency EA may also attenuate the rotational behavior of the end-stage PD models without significantly increasing the number of TH-positive dendritic fibers in the lesion-lateral SN and STR (Figures 1(a) and 1(d)). The network regulation of the central nervous system (CNS) and endocrine system may also be involved in the mechanism of EA at PD.

At present, PD is no longer believed to be a disorder that only affects the DAergic system [35, 36]. The elucidation of important gene expression profiles will enable the identification of genetic susceptibility markers, biomarkers of disease progression, and new therapeutic targets [3]. According to the standard of FCA ≥ 3 (EA versus model; log_2_ difference absolute ≥ 1), ≥70% of the genes in the cortex and STR were in profile (a) of Figure 3 (Table S11). Lesions induced by 6-OHDA may activate a compensatory cascade of neurotrophic activity within the nigrostriatal system as a physiological response to the loss of DAergic neurons in rats [39, 40]. These percentages also suggest that the effect of EA in 6-OHDA lesioned animals may result in a return of the partial gene expression levels to those of control values. FCA ≥ 3 transcripts that can be searched for in the literature were listed in Table S13. References to the functions of these transcripts in the supplementary literature 1–4 indicate that the stable expression of these transcripts is important for homeostasis.

In addition, several of the transcripts that were significantly regulated only by EA were particularly noteworthy (Table S6, profile (d) in Figure 3). EA at 100 Hz upregulated the functions of “epidermal growth factor receptor binding” in the cortex and of “angiogenesis or vasculature development” in the STR (*P* ≤ 0.01). AREG and EPGN participated in the function of epidermal growth factor receptor binding. TBX4, ANGPT2, and VEGFA (Table 3) participated in angiogenesis or vasculature development. The concrete functions of these transcripts are shown in Table S13 referred to the supplementary literature 5–9. These genes that were upregulated by EA (Table S6) were extremely important for angiogenesis or vasculature development, which contributes to the delivery of important signal molecules from the cortex to the STR or other regions.

After the relative expression level, which is either up- or downregulated, was quantified, the altered transcripts were applied to a bioinformatics analysis using the program “pathway” (Section 2). Ten pathways in the cortex and nine pathways in the STR were predominantly affected by 100 Hz EA (Section 3). The most commonly regulated pathways between the two encephalic regions included the olfactory transduction pathway, neuroactive ligand-receptor interaction pathway, amyotrophic lateral sclerosis (ALS) pathway, and long-term depression pathway. PD is a neurodegenerative disease characterized by the selective degeneration of DAergic cells in the SN and the nigrostriatal pathway. In the 6-OHDA-induced PD rat model, cotransplantation of fetal ventral mesencephalic cells with olfactory ensheathing cells (OECs) has been reported to enhance DA neuron survival, striatal reinnervation, and functional recovery [41]. OECs constitute a unique population of glial cells that accompany and ensheath the primary olfactory axons. These cells are believed to be critical for the spontaneous growth of olfactory axons within the developing and adult olfactory nervous system and have recently emerged as potential candidates for the cell-mediated repair of neural injuries [42]. However, the relationship between the olfactory transduction pathway and OECs has not yet been identified. Another important regulated pathway by EA was the neuroactive ligand-receptor interaction pathway. The signal connection was significantly enhanced in the cortex and in the lesion-lateral STR. The pathway analysis for the differential regulated genes and the functional annotation for the differential transcripts in the regulated profiles by 100 Hz EA suggest that EA functions through network regulation. In addition, we selected several transcripts in the commonly regulated pathways to identify their expressions using QRT-PCR (Figure 4(c)). Several concrete functions of these transcripts are shown on Table S13 referred to the literature 10–13. From the corresponding literature, pathways related to hormone levels and inflammatory stimuli may be targets of EA at 100 Hz.

**Figure 6:**
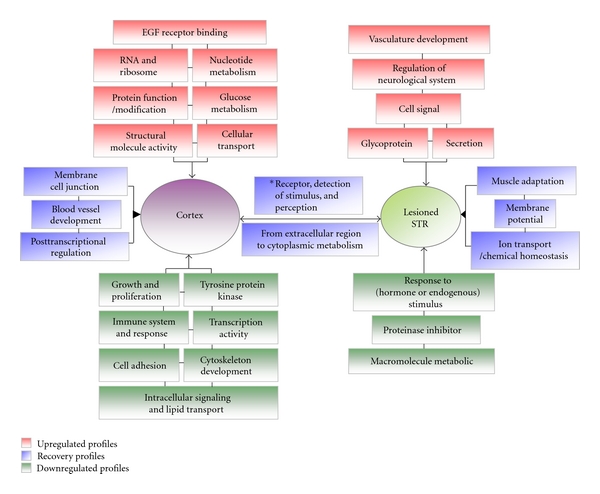
A scheme of functional annotation for transcripts in the 16 types of profiles (for discussion). There were 16 types of profiles for the differential transcripts in the three experimental groups of the cortex or lesion-lateral STR (control, model, and EA group). These profiles can be mainly divided into 5 subgroups after 100 Hz EA treatment (Figure 3). Here, the schematic summarizes the related major functional categories of the transcripts regulated in 16 types profiles (Tables S2–8). Red represents the functional annotation for the upregulated transcripts. Green represents the functional annotation for the downregulated transcripts. Blue represents the functional annotation for the transcripts that returned the levels of gene expression to control values. *This box includes olfactory receptor activity, detection of chemical stimulus involved in sensory perception of smell, G-protein coupled receptor protein signaling pathway, cell surface receptor linked signal transduction, cognition or neurological system process, and sensory perception (Table S2).

Multiple microarray studies have compared the gene expression profiles of cells within the midbrain of normal controls with those from Parkinson's diseased brains [37, 38, 43–45]. As Section 3 presents, the differential genes were divided into 16 types of expression profiles after the STC analysis (Figure 3). Transcripts in profile (a) revealed that the effect of EA in the 6-OHDA lesioned animals is to return the levels of gene expression to control values. By summarizing the functional annotations for these transcripts in the 16 profiles (Supplementary Tables S2–8), we were able to draw a schematic to present the probable relationship between the cortex and lesion-lateral STR (Figure 6). Several important functional categories were identified in this schematic. In the mutual functional clusters in the cortex and lesion-lateral STR of profile (a), the categories of “olfactory transduction and receptor activity,” “G-protein coupled receptor protein signaling,” “cognition and sensory perception,” “neurological system process,” and “integral to membrane” were the functional categories of the mutual regulated transcripts between the cortex and STR (Table S10). The identical results of the two differential analytic process revealed that there were several functional clusters in the cortex and lesion-lateral STR that are targeted by EA at 100 Hz. In addition, the transcripts in these functional clusters returned the levels of gene expression to control values. These clusters were connected vinculums between the cortex and lesion-lateral STR.

 Although we observed a therapeutic effect of high-frequency EA, it was essential to explain the possible molecular mechanism. Previously, no detailed and complete information was available about how genes in the cortex and STR were regulated by EA to maintain homeostasis. In the present work, we observed that the transcriptional levels of many functional clusters were regulated in the cortex or lesion-lateral STR when DAergic neurons were nearly inactivated. In addition, the functions of these transcripts were found to be involved in many aspects (shown in supplementary Tables S2–8). Our results also suggest that lesions induced by 6-OHDA may activate a compensatory cascade, as described above. Previously, microglial activation was observed in the lesion-lateral SN of PD rat models, and 100 Hz EA was observed to significantly inhibit the activation of microglia in the SN [15]. In the present study, many transcripts, which can positively regulate functions related to immune system processes and immune responses, were downregulated in the model cortex and were further downregulated after EA treatment (Table S5, Figures 3(c) and 6). These findings suggest that the immune suppression signal from the cortex was present after the 6-OHDA lesion, possibly because of a compensatory cascade. However, EA was able to further enrich the suppressive signal. The relationship between the suppressive signal and the two encephalic regions needs to be investigated further.

High-frequency EA resulted in a reduction in the number of apomorphine-induced rotations. We also found that EA resulted in the up- or downregulation of several other functions in the two encephalic regions (Figure 6). Combining the functional categories for the recovery profiles, these clusters form a functional network for the cortex and lesion-lateral STR, which is regulated by 100 Hz EA. In addition, the functional category of “response to hormone or endogenous stimulus” in the lesion-lateral STR is interesting. The transcripts in this functional cluster were downregulated. The level of one transcript, prolactin (PRL), is known to be below the detection limit in PD patients [46] and was also extremely low in our results (Figure 4(c)). EA at 100 Hz was unable to upregulated the level of PRL (Table S9). However, several transcripts that respond to hormones or endogenous stimuli were downregulated. The effect mechanisms underlying the phenomenon about the downregulated transcripts need to be investigated further. Moreover, the coexpression network of the transcripts in the six recovered profiles (Figure 3(a)) of the cortex and STR that were targeted by 100 Hz EA is presented in Figure S1. and Figure 5. By carefully observing the transcripts in the networks, we identified that FAD104, OLR192, BHLHB5, OTOP1, and ENDRA are the core nodes in the targeted cortex network (degree ≥ 30). PSARL, OLR522, and SLCO4A1 are the most important nodes in the lesion-lateral STR network targeted by 100 Hz EA. The major functions of the eight transcripts are shown in Table S13 referred to the supplementary literature 14–26. Combining these figures, the network density and nodes' degrees suggested that the cortex assumes the most important role in the regulation of internal balance after EA treatment (Figure 5). In addition, the cortex may be the center that gives the regulation signal. To some extent, the most commonly presented transcripts between the cortex and STR and the profiles of these transcripts in three groups may reflect the main trends of molecular regulation in vivo after EA treatment. Furthermore, we analyzed the recovered gene rate of the mutually presented genes between the cortex and STR and the rate of the regulated transcripts (log_2_ > 1 or log_2_ < −1). Both of these rates were greater than 70% (see Section 3). A dynamic gene network including several of these genes, especially genes in profiles (a)-(A), (C), and (E), may be one part of the basic framework for high-frequency EA effects (Table S2, Figures 3, 5, and 6).

Although information at the transcriptional level does not directly correlate with the levels and functions of the corresponding proteins, transcription is the first response and defense when an organism is subjected to a stimulus. As a result, changes in global transcripts may reveal the molecular mechanism of complicated disorders after high-frequency EA treatment.

## 5. Conclusions

High-frequency EA may attenuate the rotational behavior of a PD model when there was a near complete (>90%) denervation in the lesion-lateral SN. A microarray analysis of the cortex and STR revealed that 100 Hz EA is a multitarget treatment for PD and that the curative effect may be generated through regulating targeted transcripts, pathways, and functional clusters and through the spatial network interaction of targeted transcripts in different encephalic regions. In conclusion, recovering homeostasis may be an important part of high-frequency EA mechanisms.

## Supplementary Material

Supplementary Materials include two parts: Tables (Table S1-S13) and Figure S1. Table S1 included the primers which were used in real-time PCR. The related functional categories of the transcripts regulated in 16 types profles in Figure 3 were listed in Tables S2–8. The mutual regulated transcripts in both the cortex and STR were listed in Table S9, and the mutual regulated functional categories in both the cortex and STR were listed in Table S10. If Log2 absolute (EA-Model) ≥ 1 and p < 0.05, the regulated transcripts were listed in Table S11. The results of pathway analysis for regulated transcripts by EA were listed in Table S12. And the corresponding functions of expressed products for some important transcripts (Discussion section) were listed in Table S13. The complete Dynamic Gene Network of the cortex and STR are presented in Figure S1.
Figure Legends for Supplementary Materials: Figure S1 Dynamic Gene Network for genes in profile Aa-Af of Fig.3. The Dynamic Gene Network of the cortex is presented in S1A and S1B. The blue dots indicate the differentially regulated genes. The lines show the relationships between genes. The solid line denotes positive regulation, and the dashed line denotes negative regulation. The size of the dots indicates the capability of the gene to interact with others. This capability was quantified by “degree” (refer to “Materials and Methods”). The larger the degree, the more genes interacted with the corresponding gene were and the more important this gene was in the network.
