# Supplementary material for: The Cortical and Striatal Gene Expression Profile of 100 Hz Electroacupuncture Treatment in 6-Hydroxydopamine-Induced Parkinson's Disease Model
Source: Evid Based Complement Alternat Med. 2012 Jan 26;2012:908439. doi: 10.1155/2012/908439 (PMC3272844; doi:10.1155/2012/908439)
Supplement: Supplementary file 1 — Supplementary Materials include two parts: Tables (Table S1-S13) and Figure S1. Table S1 included the primers which were used in real-time PCR. The related functional categories of the transcripts regulated in 16 types profles in Figure 3 were listed in Tables S2–8. The mutual regulated transcripts in both the cortex and STR were listed in Table S9, and the mutual regulated functional categories in both the cortex and STR were listed in Table S10. If Log2 absolute (EA-Model) ≥ 1 and p < 0.05, the regulated transcripts were listed in Table S11. The results of pathway analysis for regulated transcripts by EA were listed in Table S12. And the corresponding functions of expressed products for some important transcripts (Discussion section) were listed in Table S13. The complete Dynamic Gene Network of the cortex and STR are presented in Figure S1. Figure Legends for Supplementary Materials: Figure S1 Dynamic Gene Network for genes in profile Aa-Af of Fig.3. The Dynamic Gene Network of the cortex is presented in S1A and S1B. The blue dots indicate the differentially regulated genes. The lines show the relationships between genes. The solid line denotes positive regulation, and the dashed line denotes negative regulation. The size of the dots indicates the capability of the gene to interact with others. This capability was quantified by “degree” (refer to “Materials and Methods”). The larger the degree, the more genes interacted with the corresponding gene were and the more important this gene was in the network. [file 908439.f1.pdf]

Table S1 Gene functional annotation for up-regulated transcripts by EA (profile B-a, b and c in Fig. 3)

EA-Targeted Function	Name of sub-category	p-value	Number of transcripts
**Cortex**			
Protein function and Modification	Protein of unknown function DUF622	6.33E-05	4
	Ligase activity, forming carbon-nitrogen bonds	2.58E-04	10
	Small conjugating protein ligase activity (2)	0.003215	7
	Phosphoprotein	3.99E-04	64
	Acetylation	0.003203	32
Nucleotide and Nucleotide metabolism	GTP binding (3)	2.77E-04	13
	GTPase activity	0.005642	6
	Small GTP-binding protein	0.008774	7
	Nucleotide binding	0.004805	36
	DNA recombination and DNA repair	0.007939	5
	Purine nucleotide metabolic process	0.012207	8
	Purine nucleotide biosynthetic process	0.013449	7
Cellular transport	Cytosol	0.001331	26
	Intracellular transport	0.003014	15
	Synaptic vesicle	0.014385	5
	Establishment of protein localization	0.008444	15
	Endomembrane system	0.00868	16
	Integral to endoplasmic reticulum membrane	0.010956	4
	Zymogen granule	0.013286	3
**STR**	Repeat:TPR 10	0.011438	2
Cell signal	Glycoprotein	0.012100	14
	Signal	0.012629	12
	Negative regulation of appetite or response to nutrient levels	0.014925	2

Table S2 Gene functional annotation for down-regulated transcripts by EA (profile C-a, b and c in Fig. 3)

EA-Targeted Function	Name of sub-category	p-value	Number of transcripts
**Cortex**			
Growth and Proliferation	Regulation of growth	3.15E-04	14
	Regulation of cell proliferation	4.53E-04	22
	Regulation of organ growth	9.02E-04	5
	Organ regeneration	0.001334	6
	Regeneration	0.001347	8
	Negative regulation of smooth muscle cell proliferation	0.001359	4
	Regulation of smooth muscle cell proliferation	0.001801	6
	Positive regulation of cell proliferation	0.009080	13
	Regulation of cardiac muscle growth or regulation of heart growth	0.011956	3
	Mitotic cell cycle	0.014662	9
Development	Tissue morphogenesis	0.001449	11
	Tube development	0.008710	10
	Positive regulation of multicellular organismal process	0.008933	10
Kinase	Tyrosine protein kinase	0.001435	7
	Tyrosine protein kinase, active site	0.003738	6
	Protein tyrosine kinase activity	0.006982	7
	Kinase	0.006915	15
	Protein kinase, core	0.007412	12
	Cyclin-dependent protein kinase holoenzyme complex	0.008807	3
Immune system	Positive regulation of immune system process	0.001700	11
	Regulation of leukocyte activation	0.002042	9
	Positive regulation of lymphocyte activation	0.002505	7
	Positive regulation of alpha-beta T cell	0.002654	4

	differentiation		
	Positive regulation of T cell activation	0.003691	6
	Positive regulation of immune response	0.003872	8
	Regulation of lymphocyte activation	0.004346	8
	Regulation of T cell activation	0.005101	7
	Positive regulation of CD4-positive, alpha beta T cell differentiation	0.010056	3
	Regulation of adaptive immune response	0.012039	5
	Positive regulation of B cell activation	0.012058	4
	Immunoglobulin-like fold	0.012693	11
	Immunoglobulin subtype	0.013768	8
	T cell differentiation	0.014101	5
	Plasma membrane	0.008638	42
Cell adhesion	Cell adhesion	8.72E-04	17
	Extracellular region	0.002518	28
	Protein complex binding	0.004429	10
	Intracellular signaling cascade	0.005626	24
	Extracellular region part	0.009106	17
	Variant SH3	0.009152	4
Cell signal and Transport	Lipid transport	0.007389	7
	Lipid transporter activity	0.005452	5
	DNA binding	0.008310	31
	Src homology-3 domain	0.013131	7
	Transcription factor activity	0.010139	18
	Protein amino acid phosphorylation	0.014200	17
**STR**			
Response to stimulus	Response to endogenous stimulus	0.005533	7
	Response to hormone stimulus	0.014925	6
Proteinase	Proteinase inhibitor or bait region or alpha-2-macroglobulin	0.013300	2

Table S3 Gene functional annotation for regulated transcripts by EA (p≤1, profile E-a in Fig. 3)

EA-Targeted Function	Name of sub-category	p-value	Number of transcripts
**Cortex**			
RNA and Ribosome	Structural constituent of ribosome	1.01E-17	30
	Ribosome	1.10E-17	31
	Ribonucleoprotein complex	6.56E-16	35
	Ribosomal subunit	6.65E-04	7
	Cytosolic ribosome	0.004038	5
	Large ribosomal subunit	0.010777	4
	Ribosomal small subunit biogenesis	0.012186	3
	RNA processing	0.020564	11
Protein and protein processing	Cytoplasm	0.007598	29
	Translation	8.09E-17	34
	Protein biosynthesis	5.87E-07	12
	Translational elongation	3.46E-05	9
	Acetylation	4.06E-06	34
	Phosphoprotein	0.003938	49
	UBL conjugation	0.015003	9
Metabolism	Glyceraldehyde-3-phosphate dehydrogenase activity or Oxidoreductase activity	4.43E-05	6
	Glucose metabolic process	1.09E-04	11
	Generation of precursor metabolites and energy	0.004210	10
	NAD or NADH binding	0.001092	7
Structure	Structural molecule activity	1.88E-12	33
	Intracellular non-membrane-bounded organelle	2.52E-07	49
**STR**			
Cell Signal	Neuropeptide signaling pathway	1.69E-05	7
	Neuropeptide	1.74E-04	5
	Signal	1.77E-04	28
Secretion	Extracellular region	8.98E-04	22

	Secreted	9.94E-04	17
	Regulation of secretion	0.011457	7
	Glycoprotein	0.002002	30
Protein	Compositionally biased region:Ser-rich	0.006704	6
	Glycosylation site:N-linked (GlcNAc...)	0.008202	28
	Epidermis or Ectoderm development	0.008123	5
	Regulation of system process	0.011394	8
Development	Regulation of cellular localization	0.005438	8
	Regulation of muscle contraction	0.014215	4
	Regulation of neurological system process	0.014887	6

Table S4 Gene functional annotation for regulated transcripts by EA (p≤1, profile E-b in Fig. 3)

EA-Targeted Function	Name of sub-category	p-value	Number of transcripts
**Cortex**			
Protein	Basic leucine zipper	1.25E-04	4
	Basic-leucine zipper (bZIP) transcription factor	2.08E-04	5
	CCAAT/enhancer-binding	3.06E-04	3
	Protein dimerization activity	0.005100	10
Transcription	Transcription factor activity	5.44E-04	13
	Transcription regulator activity	5.74E-04	17
	Sequence-specific DNA binding	9.94E-04	11
	Regulation of transcription from RNA polymerase II promoter	0.002696	12
	Regulation of transcription, DNA-dependent or RNA metabolic process	0.003510	18
	DNA binding	0.007374	17
	Transcription activator activity	0.007890	7
	Transcription regulation	0.009472	11
	Transcription repressor activity	0.013137	6
Development	Positive regulation of developmental process	0.001294	9
	Regulation of cell development	0.005380	7
	Embryonic placenta development	0.006989	4
	Cytoskeleton	0.008597	3
	Positive regulation of cell differentiation	0.007651	7
	Positive regulation of macromolecule metabolic process	0.010296	13
	Positive regulation of JAK-STAT cascade	0.011649	3
Signal	Plasma membrane	0.006914	24
	Extracellular region	0.014396	15
	Immune response	0.007403	9
**STR**			
Cell Signal	Response to hormone or endogenous stimulus	0.001222	10

	Cellular response to hormone stimulus	0.006199	5
	Plasma membrane	0.001697	23
	Plasma membrane part	0.005853	15
	Phospholipid transport or lipid transporter activity	0.011272	3
	Response to organic substance	0.007872	12
	Regulation of ossification	0.008328	4
Development	Positive regulation of macromolecule metabolic process	0.009762	11

Table S5 Primers used in real-time PCR

Gene name	GenBank _ID	PCR fragment	Sequence of primers
GRIA2	NM_017261.2	nt2687-2768	5'-TACGGCATCGCCACACCTAA -3'
			5'-AGACGCCTTGCTCACTGAGTTTC -3'
NOS3	NM_021838.2	nt1064-1157	5'-GCGGCTGGTACATGAGTTCAGA-3'
			5'-AGATCCATGCAGACAGCCACA-3'
HRH4	NM_131909.1	nt325-504	5'-TGTGCACAGCATCCGTCTACAG-3'
			5'-AAGAATCCGAAGCCAGAATCATTG-3'
PRL	NM_012629.1	nt399-538	5'-CCAAGATCGTGAGTTTATTGCCAAG-3'
			5'-TCCAGGAGTGCACCAAACTGAG-3'
OPRK1	NM_017167.2	nt609-704	5’-TCTGCATTTGGCTACTGGCATC-3’
			5’-AGGAGCATTCAATGACATCCACA-3’
OLR1078	NM_207597.1	nt281-382	5’-ATGCAGGATGCATTACCCAGA-3’
			5’-GACAGATGGCTACGTAACGGTCATA-3’
OLR1523	NM_001000713.1	nt803-896	5’-ACAAAGCCGTGTCCATCAGCTAC-3’
			5’-TTCATGGCTGCCTGCATCTC-3’
PLA2G10	NM_017176.1	nt173-344	5’-CTCGATCGCCGATGGCTTA-3’
			5’-TCCATGCACTTCCACGGGTA-3’

Figure Legends for Supplementary Materials

Figure S1 Dynamic Gene Network for genes in profile Aa-Af of Fig.3. The Dynamic Gene Network of the cortex is presented in S1A and S1B. The blue dots indicate the differentially regulated genes. The lines show the relationships between genes. The solid line denotes positive regulation, and the dashed line denotes negative regulation. The size of the dots indicates the capability of the gene to interact with others. This capability was quantified by "degree" (refer to "Materials and Methods"). The larger the degree, the more genes interacted with the corresponding gene were and the more important this gene was in the network.

Figure S1A

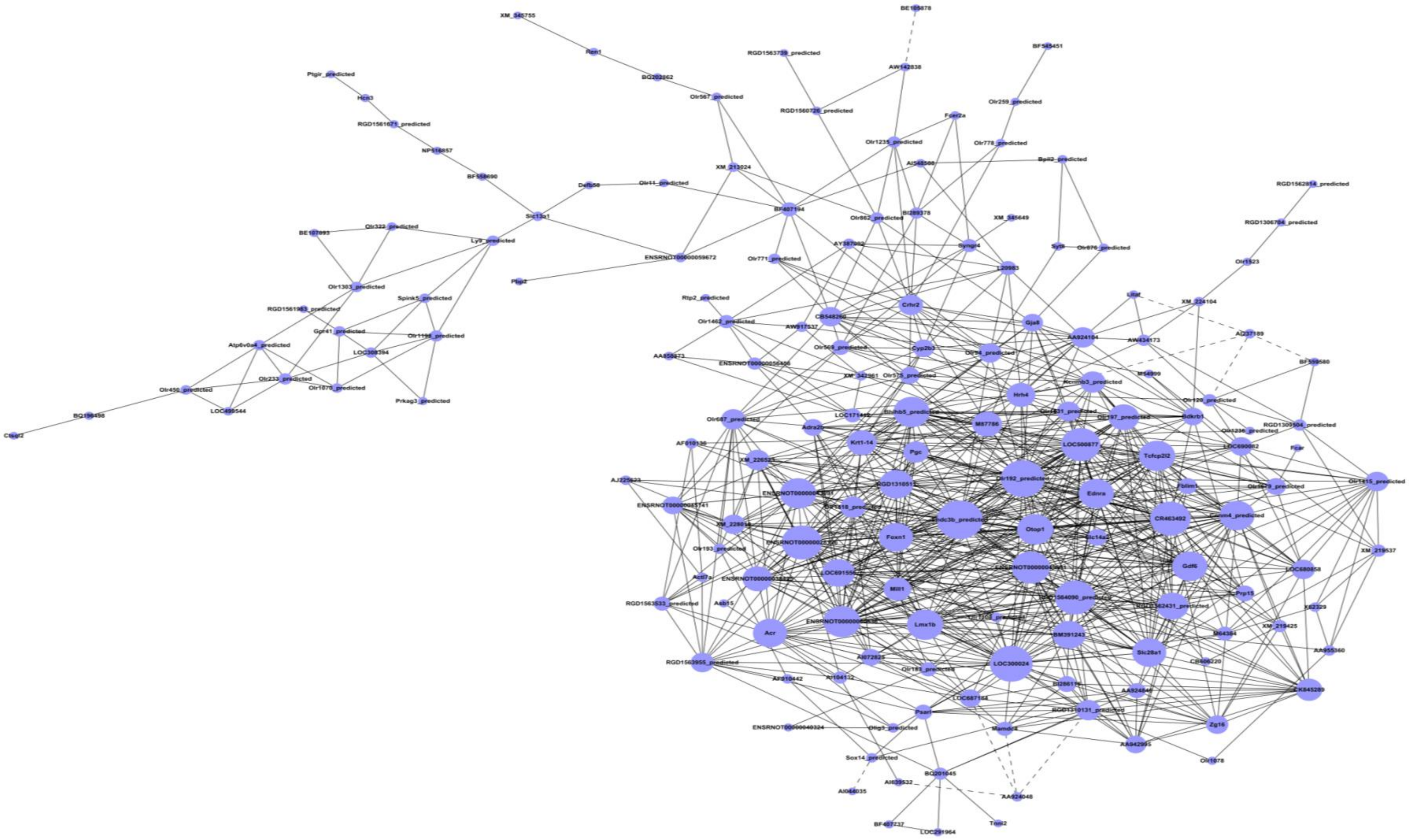

XM_345755
Ren1
BQ202862
Olr567_predicted
Ptgir_predicted
Hcn3
RGD1561671_predicted
NP516857
BF558690
Slc13a1
Defb50
Olr11_predicted
XM_213024
BF407194
ENSRNOT00000059672
Ptp2
BE109893
Olr322_predicted
Ly9_predicted
Olr1303_predicted
Spink5_predicted
RGD1561983_predicted
Gpr41_predicted
Olr1196_predicted
Atp6v0a4_predicted
LOC308394
Olr233_predicted
Olr1070_predicted
Olr450_predicted
LOC499544
Prkag3_predicted
BQ196498
Cbsd2
BE109878
RGD1563739_predicted
AW142838
BF545451
RGD1560726_predicted
Fcer2a
Olr259_predicted
Olr1235_predicted
Olr778_predicted
Bpil2_predicted
Olr862_predicted
XM_345649
Syngr4
Olr876_predicted
Olr771_predicted
RGD1562814_predicted
RGD1306704_predicted
Olr1523
XM_224104
Rtp2_predicted
Olr1462_predicted
Crhr2
Gja8
AA924104
AW434173
AI237189
BF559580
Olr569_predicted
Cyp2b3
ENSRNOT00000055406
Hrh4
Olr129_predicted
Olr687_predicted
LOC171440
Bhlhb5_predicted
M87786
Olr197_predicted
Bdkrb1
RGD1309504_predicted
Adra2c
Krt1-14
Pgc
LOC500877
Tcfcp2l2
Fcer
XM_226525
RGD1310511
Olr192_predicted
Ednra
Fblim1
Olr1415_predicted
ENSRNOT00000031141
XM_228016
Sdc3b_predicted
Otop1
CR463492
Foxn1
Slc14a2
Olr193_predicted
Gdf6
LOC680858
XM_219537
LOC691556
Mll1
Prp15
RGD1563533_predicted
Asb15
Acr
Lmx1b
BM391243
XM_219425
AA955360
Slc28a1
RGD1563955_predicted
AI072825
Olr193_predicted
LOC300024
CB606220
AA924846
CK845289
AF010442
AI104132
LOC687184
Psarl
RGD1310131_predicted
Zg16
ENSRNOT00000040324
Olig3_predicted
Mamdc4
AA942995
Olr1078
Sox14_predicted
BQ201645
AI064035
AA924048
BF407237
LOC291964
Tnni2

Figure S1B

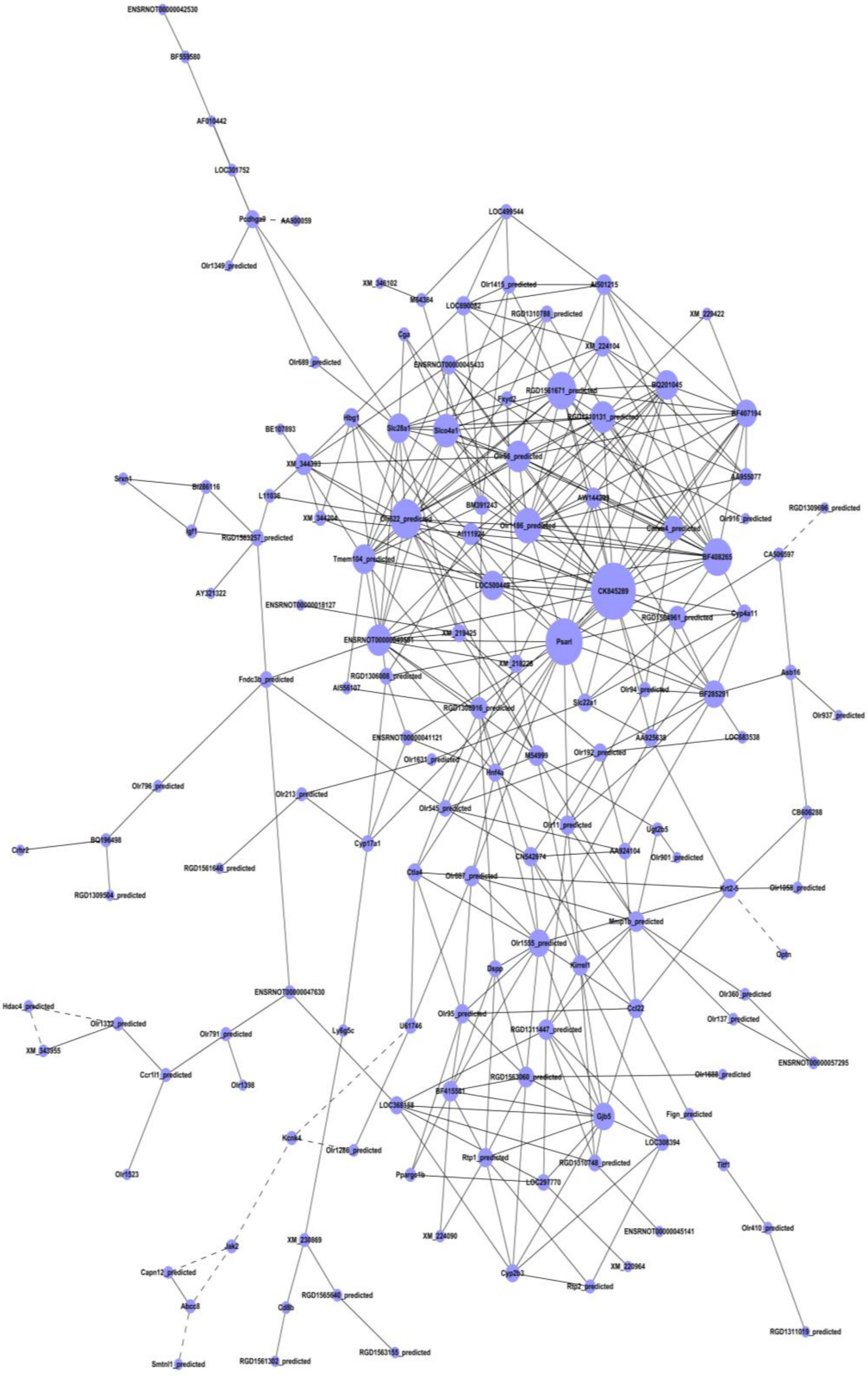
ENSRNOT00000042530
BF559580
AF010442
LOC301752
Pcdhga9
AA800059
Olr1349_predicted
LOC499544
XM_346102
M64384
Olr1415_predicted
AI501215
LOC690092
RGD1310788_predicted
XM_229422
Cpa
XM_224104
ENSRNOT00000045433
Olr689_predicted
RGD1561671_predicted
BQ201045
Fxyd2
Hbg1
Slc28a1
Slco4a1
RGD1310131_predicted
BF407194
BE107893
XM_344393
Olr56_predicted
AA955077
Srxn1
BI286116
L11036
AW144299
BM391243
RGD1309696_predicted
XM_344204
Olr622_predicted
Olr1186_predicted
Olr916_predicted
Igf1
RGD1563257_predicted
Tmem104_predicted
BF408265
CA506597
AY321322
CK845289
ENSRNOT00000018127
RGD1564961_predicted
Cyp4a11
XM_219425
Psarl
XM_218226
Fndc3b_predicted
RGD1306008_predicted
AI556107
Olr94_predicted
BF285291
Asb16
Slc22a1
RGD1309916_predicted
Olr937_predicted
ENSRNOT00000041121
AA925638
LOC683538
Olr1633_predicted
M54999
Olr192_predicted
Hnf4a
Olr796_predicted
Olr213_predicted
Olr545_predicted
CB606288
Olr11_predicted
Ugt2b5
BQ196498
Cyp17a1
CN542674
AA924104
Olr901_predicted
RGD1561646_predicted
Ctla4
Olr687_predicted
Krt2-5
Olr1058_predicted
RGD1309504_predicted
Mmp1b_predicted
Olr1555_predicted
Dspp
Kirrel1
Optn
ENSRNOT00000047630
Hdac4_predicted
Olr1332_predicted
Olr95_predicted
Ccl22
Olr360_predicted
Olr137_predicted
Ly6g5c
U61746
Olr791_predicted
RGD1311447_predicted
XM_342955
Ccr1l1_predicted
Olr1398
RGD1563060_predicted
Olr1686_predicted
ENSRNOT00000057295
BF415581
LOC368158
Gjb5
Fign_predicted
Kcnk4
Olr1286_predicted
LOC308394
Rtp1_predicted
RGD1310748_predicted
Olr1523
Ppargc1b
LOC297770
ENSRNOT00000045141
Olr410_predicted
Jak2
XM_230869
XM_224090
Capn12_predicted
XM_220964
Cyp2b3
Rtp2_predicted
Abcc8
Cd8b
RGD1565640_predicted
RGD1563155_predicted
Smtnl1_predicted
RGD1561302_predicted
RGD1311019_predicted
